# Evaluating the Impact of Financial Navigation on Financial Catastrophe and Distress for Cancer Care: A Randomized Control Trial- COST-FIN

**DOI:** 10.21203/rs.3.rs-7686399/v1

**Published:** 2026-01-21

**Authors:** Amir H. Sohail, Oluwasegun Afolaranmi, Funmilola Olanike Wuraola, Matthew Caputo, George Guiterrez, Adewale Oyewole, Dorcas O. Ebekue, Zainab Oyindamola Adegbite, Clement Awe, Titilope Ogunniyi, Akinlusi Opeyemi, Chinyere Nwankwo, Frances Uwechue, Dan Sherman, Clara N. Lambert, Joseph Adedayo, Kristina Diaz, Elizabeth Nicole Christian, Fatimah Abdulkareem, Olufemi Akin-Adigun, Adewunmi Akingbola, Sophia Okeke, Gregory Knapp, Anna Dare, Toluwanimi Aduloju, Moyinoluwa Akinwumi, Ciaran Navin Kohli-Lynch, Ruohui Chen, Chukwumere Nwogu, Lisa R Hirschhorn, Andrei Adin-Cristian, Chinenye Iwuji, Oge Ilegbune, Mutiu Jimoh, Bindiya Chugani, Olise Oputa, Shilpa Murthy, Dustin French, Ron Ackermann, Robert Murphy, Anthony Seddoh, Peter Kingham, Olusegun Isaac Alatise, Juliet S Lumati

**Affiliations:** University of New Mexico - Albuquerque: The University of New Mexico; Mayo Clinic Rochester: Mayo Clinic Minnesota; Obafemi Awolowo University Faculty of Clinical Sciences; University of Minnesota School of Public Health: University of Minnesota Twin Cities School of Public Health; Northwestern University; Obafemi Awolowo University; Obafemi Awolowo University Teaching Hospital Complex; Lakeshore Cancer Center, Lagos, Nigeria; Lakeshore Cancer Center, Lagos Nigeria; Obafemi Awolowo University Teaching Hospital Complex; Obafemi Awolowo University Teaching Hospital Complex; Lakeshore Cancer Center, Lagos Nigeria; University at Buffalo; The NaVectis Group; TailorMed Financial Navigation Platform; Lagos University Teaching Hospital; Northwestern University Feinberg School of Medicine; Northwestern University; University of Lagos College of Medicine; University of Oxford Department of Oncology; University of Cambridge; University of Ibadan College of Medicine; Dalhousie University Department of Surgery; University of Toronto; Lakeshore Cancer Center, Lagos, Nigeria; Lakeshore Cancer Center, Lagos, Nigeria; Northwestern University - Chicago; Northwestern University Department of Preventive Medicine; Roswell Park Cancer Institute: Roswell Park Comprehensive Cancer Center; Northwestern University; Northwestern University Department of Preventive Medicine; Lakeshore Cancer Center, Lagos, Nigeria; Lakeshore Cancer Center, Lagos, Nigeria; Lakeshore Cancer Center, Lagos, Nigeria; Lakeshore Cancer Center, Lagos, Nigeria; Northwestern University; Yale University Department of Surgery; Northwestern University Feinberg School of Medicine; Northwestern University; Northwestern University; World Bank Group; Memorial Sloan Kettering Cancer Center; Obafemi Awolowo University Teaching Hospital Complex; Northwestern University Feinberg School of Medicine

**Keywords:** Financial navigation, financial toxicity, cancer, financial distress, financial catastrophe, treatment adherence, Sub-Saharan Africa

## Abstract

**Background:**

As with most Sub-Saharan African countries, Nigeria has a rising incidence of cancer, with disproportionate mortality rates. The financial burden of cancer care often results in catastrophic healthcare spending, leading to treatment refusal, disruption, and discontinuation. This is particularly significant in Nigeria, where nearly all patients are uninsured, and out-of-pocket costs often exceed households’ ability to pay. Financial Navigation Programs (FNPs) have been shown to mitigate treatment-related financial toxicity in cancer care and reduce treatment abandonment, but there is a paucity of high-quality data on this intervention in resource-constrained settings. Here, we present a randomized controlled trial to evaluate the impact of a novel FNP in Nigeria.

**Methods:**

We designed the COST-FIN trial, a multi-site pragmatic single-blinded randomized controlled trial of newly diagnosed (<6 weeks from diagnosis) adults (≥18 years) with breast, colorectal, or prostate cancer at two tertiary cancer centers in Nigeria. Participants (n=200) will be randomized (1:1) to either the intervention (FNP) or the control arm and followed for 12 months. Data on key individual, treatment, and financial parameters will be collected via structured interviews and chart abstraction at baseline, 3, 6, and 12-month follow-up. In addition, participants randomized to the FNP will receive a tailored financial literacy assessment, financial planning support, and enhanced access to resources by trained financial navigators. Primary and secondary outcomes are Financial Catastrophe (FC) and Financial Distress (FD), respectively. Exploratory outcomes will include cost-related non-adherence and cost-effectiveness of the program. An interim analysis will be conducted when 50% of the estimated accruals reach 6 months of follow-up, with crossover if compelling evidence of benefit is demonstrated at that time point. All participants will be followed for 12 months from recruitment.

**Discussion:**

This first-of-its-kind study will provide evidence on the role of FNP in potentially eliminating financial barriers to cancer care in Nigeria. Given the country’s renewed interest in cancer control through the passage of the National Cancer Control Plan, findings from this study have the potential to influence policy reform and set the stage for further studies to evaluate the scalability and implementation of similar interventions in resource-limited settings.

**Trial registration:**

ClinicalTrials.gov NCT06630962. Oct 8, 2024 (https://clinicaltrials.gov/study/NCT06630962)

## BACKGROUND AND RATIONALE {6}

Cancer disproportionately affects individuals in low- and middle-income countries (LMICs)^1^, which are projected to account for approximately 75% of new cancer cases and 65% of cancer-related deaths globally by 2030.^2^ Sub-Saharan Africa experiences the highest rate of premature cancer-related mortality, and in Nigeria – the region’s most populous nation – over 100,000 new cancer cases are diagnosed annually. Amongst the most common are breast, prostate, and colorectal cancer.^1–3^ Cancer survival outcomes among Nigerian patients are poor, with an overall 5-year survival estimated to be less than 50%,^4–10^ significantly lower than the 90% survival rate observed in high-income countries. This disparity is driven by various factors, including a lack of universal screening programs, inadequate healthcare financing, and ineffective health systems.^11–14^ Nigeria ranks below the 30th percentile for effective Universal Health Coverage, and ineffective health systems are associated with increased risk of cancer-specific mortality.^15,16^

The lack of universal cancer screening programs and poverty significantly influence patterns of healthcare access and delivery.^17^ In Nigeria and similar Sub-Saharan African countries, over 80% of cancer patients present with advanced-stage disease. Financial barriers – particularly the inability to afford early diagnostic services – account for an estimated 60–70% of these delays in presentation and treatment initiation.^18^

Nigeria has undertaken concerted efforts to improve cancer prevention, control, and treatment in recent years with the establishment of the National Cancer Control Plan (2018–2022), a $300 billion investment to centralize and provide multidisciplinary cancer care at existing facilities.^19^ However, despite these developments, access to cancer care is unlikely to expand in Nigeria without significant improvement in financial risk protection since an estimated 40% of Nigerians live below the international poverty line of 2.15 USD per day.^20^ Furthermore, our prior studies on estimates of out-of-pocket costs for cancer treatment in Nigeria show that these costs can exceed the per capita Gross Domestic Product (GDP) of the country.^21–24^

The National Health Insurance Scheme (NHIS) – a government-sponsored initiative launched in 2003 to provide financial access to quality healthcare for all Nigerians – has not achieved its goal of reducing the risk of financial toxicity for cancer patients.^25^ This gap in effective cancer care delivery is multifactorial, driven by low public health insurance enrollment (less than 5% of the population), suboptimal public investment, and inefficient health service delivery mechanisms.^25^

Therefore, as a result of inadequate financial protection, the majority of cancer patients in Nigeria either forgo care or have to self-navigate household resources, which results in significant consequences for their economic well-being. A 2019 study by Nnabugwu *et al*. found that among a cohort of 306 surgical cancer patients in Nigeria, 99% relied on personal savings, and 43% resorted to borrowing to pay for treatment costs.^26^ Similarly, data on breast cancer patients published by our co-investigators at the Obafemi Awolowo University Teaching Hospital (OAUTHC) demonstrated that 72% of households borrowed money, 20% experienced job loss, and 9% had to interrupt their children’s education to finance cancer treatment.^27^ Our studies at Lakeshore Cancer Center (LCC) – a private facility in Lagos State, Nigeria – found that all colorectal cancer patients faced a high risk of financial catastrophe (FC), while three-quarters (74%) of breast cancer patients also risked FC, despite only 30% completing treatment.^28,29^

In addition to the risk of impoverishment, FC also results in worse survival outcomes due to delayed treatment initiation, poor adherence, and increased loss-to-follow-up rates.^17,30–32^ Delays and disruptions in cancer care have been consistently associated with increased cancer-specific mortality across multiple cancer types and health systems in both high and low-income settings.^33–35^ A systematic review of 319 Nigerian breast cancer patients demonstrated that greater than 50% did not complete chemotherapy or received doses at irregular intervals, and utilization of multimodal treatments was severely limited.^30^ Further, data from cancer patients treated at OAUTHC showed that 30–40% declined the recommended therapies due to costs.^27,36^ Similarly, at LCC, nearly 70% of patients with a new cancer diagnosis did not complete treatment.^31^ Finally, the African Breast Cancer – Disparities in Outcomes (ABC-DO) study, a prospective cohort study investigating survival after breast cancer in five countries in Sub-Saharan Africa, found that 47% of patients did not undergo curative surgery due to the burden of out-of-pocket costs, with Nigeria representing the highest number of untreated patients.^37^

## INNOVATION

### Financial Navigation Programs (FNPs) in Cancer Care {6b}

#### Impact on out-of-pocket costs

FNPs are structured interventions designed to proactively address the cost-related challenges of cancer care. They have been demonstrated to reduce financial toxicity, relieve cost-related anxiety, and provide concrete assistance with medical and non-medical costs.^38–39^ In fact, FNPs are now the standard of care for National Cancer Institute (NCI) designated cancer centers in the United States. In a study involving 274 NCI Community Oncology Research Program practices in the United States, 96% offered financial assistance to help patients pay for their treatment.^40^

Further, these programs have been shown to result in significant cost savings not only for patients but also for healthcare institutions. In a multi-site study involving four hospitals with NaVectis-trained financial navigators, institutions collectively secured an average of $3.5 million annually in financial assistance for oncology patients.^41^ This translated to $2.1 million in annual savings through reductions in charity care and write-offs. Among the 3600 patients served, approximately one-third (32%) received some form of financial support, with the average savings per patient being $35,294 through premium assistance, $12,256 via insurance enrollment, $33,265 from access to free medications, $3076 in copay assistance, and $880 from community-based support programs.^41^ These findings highlight the scalable financial impact of structured FNPs – not only in alleviating patient financial burden but also in improving institutional sustainability and resource stewardship.

#### Impact on Clinical Outcomes

Beyond financial relief, FNPs have been increasingly associated with meaningful improvements in clinical outcomes. Structured financial navigation has been shown to reduce delays in chemotherapy initiation, minimize interruptions in care, and lower rates of treatment abandonment – particularly among socioeconomically vulnerable populations.^41^ In a multi-site evaluation of over 1000 patients at community oncology practices conducted by TailorMed, FNPs were associated with significantly reduced treatment abandonment and improved time to treatment initiation. Patients receiving financial navigation had a 37% higher likelihood of initiating treatment within guideline-recommended timeframes and reported fewer delays in accessing care.

In addition to improving treatment adherence, FNPs have been linked to reductions in avoidable emergency department visits and hospitalizations, earlier engagement in care, and improved care coordination. Patients supported by financial navigators are also more likely to access supportive services, including palliative care, nutritional counseling, and mental health resources, thereby enhancing symptom management and overall quality of life. While survival data are still emerging, earlier initiation and continuity of treatment – facilitated by robust financial navigation – have been associated with improved cancer-specific survival outcomes in select patient populations. Thus, collectively, these findings support the inclusion of FNPs as a standard component of comprehensive cancer care delivery.^38,41,42^

### Crucial Gaps in Evidence on Impact of Financial Navigation in Sub-Saharan Africa

It is noteworthy that although FNPs have been extensively studied in the United States – through rigorous trials such as CAFÉ,^43^ LIFT,^44^ COSTCOM ^45^, FINassist,^46^ CC Links,^47^ and HINT ^48^ – there is a paucity of high-quality randomized evidence evaluating FN effectiveness in LMICs. Evaluating FNPs in LMIC contexts is essential to understanding their feasibility, effectiveness, and equity impact in settings where financial barriers are most pronounced. To date, no randomized controlled trial has examined the implementation or impact of FNPs specifically in Sub-Saharan Africa. This represents a critical evidence gap, particularly given the region’s large cancer burden and high rates of out-of-pocket expenditure, treatment abandonment, FC, and FD associated with cancer care.

### Preliminary Data from Nigeria on the Feasibility of Establishing an FNP

Preliminary data to assess the feasibility of a randomized controlled trial investigating the impact of an FNP in cancer care were collected at two major cancer centers in Nigeria: LCC and OAUTHC. This feasibility assessment facilitated a wide array of public- and private-sector partner engagement and perspective assessment. At LCC, a baseline feasibility survey of 19 stakeholders using the implementation science RE-AIMs Framework showed that 95% agreed or strongly agreed that an FNP would be beneficial, 95% liked the idea, and 90% agreed or strongly agreed that an FNP would be suitable. Among providers, 83% agreed or strongly agreed that assigning a financial navigator was a good match for patient needs, and 90% believed it was feasible. Similarly, at OAUTHC, where nine stakeholders responded, 90% agreed or strongly agreed that an FNP would be beneficial, and 90% welcomed the idea as feasible. Of note, LCC leadership perceived the benefits of an FNP to be high and permanently hired a financial navigator on staff before the inception of this study.

## OBJECTIVE AND SPECIFIC AIMS {7}

### The objective of this study is to generate novel evidence on the effectiveness of an FNP in Sub-Saharan Africa by evaluating whether FNPs can improve access to multidisciplinary cancer treatment and reduce the financial toxicity from cancer treatment.

By assessing both patient-level and system-level outcomes, this study aims to establish whether structured financial navigation support can mitigate the economic burden of cancer care and promote treatment adherence in resource-limited settings.

### Measures of Financial Toxicity

#### • Financial Catastrophe (FC)

According to the World Health Organization (WHO), FC is defined as out-of-pocket healthcare expenses that exceed one or more of the following thresholds: 10% of household income (HHI), 25% of total household expenditure (HHE), or 40% of non-subsistence expenditure (HSE).^49^

#### • Financial Distress (FD)

FD is measured by the Comprehensive Score for Financial Toxicity-Functional Assessment of Chronic Illness Therapy (COST-FACIT) tool, a validated tool for this purpose amongst individuals with cancer.^50^ COST-FACIT comprises an 11-item questionnaire, each rated on a 5-point Likert scale, and is designed to measure financial burden experienced by patients due to their illness or treatment, with lower scores representing worse financial toxicity. This tool has been adapted and validated in several countries, including Nigeria, to ensure cultural and contextual relevance.

## SPECIFIC AIMS OF THE TRIAL

The aims of this study are as follows (Table 1):

1. To evaluate the effect of the FNP on FC in patients with breast, prostate, and colorectal cancer.
2. To determine whether the FNP reduces FD for patients with breast, prostate, and colorectal cancer.
3. To determine the association of FNP with cost-related nonadherence to cancer care for patients with breast, prostate, and colorectal cancer.
4. To evaluate the cost-effectiveness of the implementation of an FNP in Nigeria

We hypothesize that the implementation of an FNP will not only be associated with reduced financial toxicity and improved adherence to cancer treatment but will also prove to be a cost-effective intervention in this resource-limited setting. This hypothesis reflects the potential for FNPs to deliver both clinical and economic value in LMICs, where patients face disproportionately high out-of-pocket costs and health systems operate with constrained financial resources.

## METHODS

### Trial Design {8}

**Figure 1:**
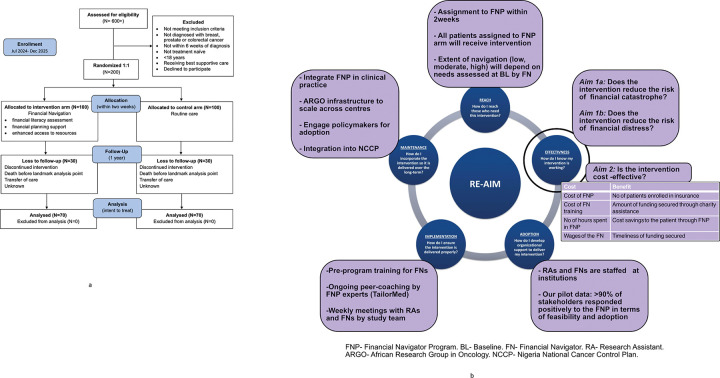
a: PRISMA Flow Diagram for COST-FIN Study Design b: Detailed Framework of COST-FIN Study

**Figure 2:**
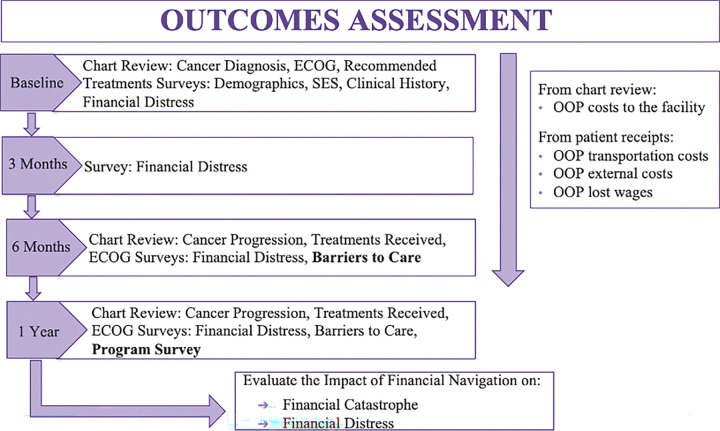
Outcomes Assessment Protocol for COST-FIN Study

A prospective single-blinded randomized controlled trial of newly diagnosed breast, prostate, or colorectal cancer patients will be conducted at two sites in Nigeria, namely, LCC and OAUTHC. At each site and for each cancer type/stage, participants will be randomized 1:1 to either the intervention arm (FNP) or the control arm (no FNP) and subsequently followed for up to 12 months (Figures 1a and 1b). Data on key parameters will be collected via structured interviews at baseline, 3 months, 6 months, and 12 months post-randomization (Figure 2). Participation in the study will require frequent telephone communication, and therefore, telephone transfer credit will be provided to all participants. Research Electronic Data Capture (REDCap) will be used for data collection, with all participant consents, surveys, clinical data, and cost receipts entered.^51^ The REDCap will be hosted at OAUTHC.

### Institutional Board Review Approval

The study has been approved by the Northwestern University Institutional Review Board (IRB), including all informed consent documents, patient materials, and study brochures. Institutional approval has also been granted by local review boards in Lagos (LCC) and OAUTHC. All study personnel will maintain active certification in human subjects’ protection.

### Study Setting and Participant Selection

#### Study Sites {9 and 15}

The study will be conducted at two sites:

1. LCC (a private facility) in Lagos State, Nigeria
2. OAUTHC (a public tertiary healthcare facility) in Ile-Ife, Osun State, Nigeria

### Both participating institutions serve as major tertiary referral centers in Nigeria and regularly receive patients from across the West African subregion, facilitating rapid and diverse participant recruitment.

Together, the two centers maintain a combined cancer registry with over 3,000 patients as of June 2025 and prospectively enroll new cases, ensuring a robust pipeline of eligible participants. **LCC** is Nigeria’s first private comprehensive cancer center, established in partnership with Roswell Park Cancer Institute in the U.S., and provides multidisciplinary oncology care in an urban setting. **OAUTHC** is a leading public-sector academic institution and a founding member of the African Research Group for Oncology (**ARGO)** consortium. Both institutions are active ARGO members, reflecting their longstanding commitment to collaborative oncology research, data quality, and implementation science.

Furthermore, conducting the trial across both a private (LCC) and a public (OAUTHC) facility promotes **participant heterogeneity. It enhances the generalizability** of the findings to a range of institutional and healthcare system contexts – a crucial consideration for future national or regional scale-up of FNPs.

#### The African Research Group for Oncology

Established in 2013, the African Research Group for Oncology (ARGO) is a National Cancer Institute-recognized consortium that enhances cancer research capabilities through access to cutting-edge technologies, advanced research methodologies, and expert knowledge.52 The consortium includes 31 medical institutions across Nigeria and four from the U.S. and Canada, and regularly collaborates with MSKCC, Albert Einstein College of Medicine, and Northwestern University. It aims to promote collaboration and generate high-quality evidence to inform regional clinical management guidelines, develop prevention and early detection strategies, expand access to universal cancer care, and strengthen cancer care training in rural and underserved communities across Nigeria.^52^ From a research standpoint, employing a multidisciplinary approach and drawing on expertise from world-leading oncologists, geneticists, epidemiologists, and public health practitioners, ARGO fosters comprehensive research initiatives that address the entire continuum of cancer care. Since its establishment, ARGO has launched 38 multi-institutional cancer research projects across Nigeria in collaboration with its partner institutions, addressing an array of dimensions to cancer care, from early detection and diagnosis to treatment and survivorship.^52^

#### Inclusion Criteria {10}

1. Age >18 years
2. New diagnosis of breast, prostate, or colorectal cancer
3. Presentation at study site within six weeks of diagnosis
4. Treatment naive
5. Candidate for treatment (not receiving best supportive care)

#### Exclusion Criteria

1. Age <18 years
2. Participants unable to comply with follow-up
3. Patients already undergoing cancer treatment
4. Diagnosed >6 weeks before assessment for study enrollment.
5. Recurrent breast, prostate, or colorectal cancer
6. Patients with cancer types other than breast, prostate, or colorectal cancer

### Details of Intervention - Role of Financial Navigators, Participant Incentives, and Key Program Features {11a, 11b, 11c, 11d}

#### Services Provided by Financial Navigators:

The FNP will provide patients at each site with access to a dedicated financial navigator. Services provided by financial navigators will include:

##### 1. Patient Financial Education:

Deliver structured education sessions to improve patients’ financial literacy, with a focus on understanding healthcare costs, budgeting, and strategies for minimizing out-of-pocket expenses during cancer treatment.

##### 2. Personalized Financial Planning:

Support patients in developing individualized financial plans based on their household income, debt burden, and available assets. Plans will include cost forecasting for diagnostics and treatment, strategies for avoiding FC, and behavioral tools for improving financial stability.

##### 3. Resource Navigation and Access:

Provide patients with enhanced access to external financial resources, including enrollment into insurance, charitable foundations, government aid programs, drug co-pay assistance, hospital-based relief funds, and community-based financial support services.

#### Individualized Financial Navigation Tailored to Patient Needs

**Figure 3:**
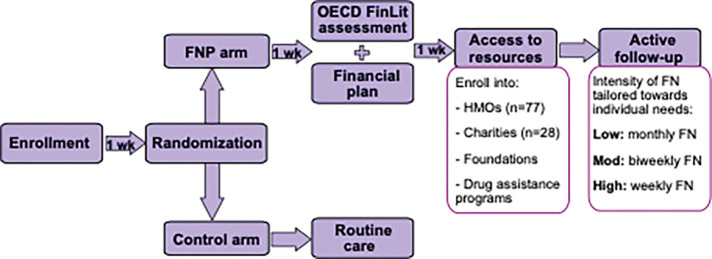
Description of Financial Navigation and Participant Incentives in COST-FIN

The intensity and frequency of financial navigation services will be customized based on each patient’s economic circumstances and level of financial vulnerability. Upon enrollment, financial navigators will assess the patient’s financial profile – including income, debt, insurance status, and treatment plan – and use this information to develop a personalized communication and intervention plan. Patients with higher risk of FC (e.g., uninsured, late-stage disease, or high anticipated out-of-pocket costs) will receive more intense navigation, including more frequent follow-up, active facilitation of applications for support programs, and detailed financial planning. Patients with fewer needs may require only limited follow-up or targeted assistance with insurance enrollment. Regardless of navigation intensity, all patients will be followed for 12 months post-randomization to assess both short- and long-term outcomes, including financial toxicity (FC and FD) and care adherence. (Figure 3)

#### Financial Navigation Program Oversight and Implementation

The FNP will be implemented in partnership with two established organizations – TailorMed and NaVectis Group – that specialize in financial navigation training and operational optimization for healthcare systems.^53,54^ These organizations have extensive experience supporting hospitals and cancer centers in building sustainable financial navigation infrastructures. Both TailorMed and NaVectis offer structured, evidence-based training programs for healthcare providers, focused on screening for FD, streamlining access to financial aid, and maximizing the efficiency of institutional workflows. Their interventions have been shown to significantly reduce patient FD and reduce treatment abandonment while also decreasing uncompensated care and financial losses for institutions.^41^ Leveraging their expertise will ensure high-fidelity implementation, capacity building for local teams, and potential for scale-up across other oncology centers in Nigeria and Sub-Saharan Africa.

#### Training and Support for Financial Navigators and Research Staff

##### Financial Navigators

Financial navigators will undergo a three-month pre-implementation phase focused on resource mapping and capacity building prior to trial enrollment. During this period, financial navigators will systematically identify and compile a comprehensive database of all available funding sources for cancer patients in Nigeria, including local, national, and international charitable organizations, public-sector assistance programs, pharmaceutical aid, and insurance options. Each resource will be cataloged with its corresponding eligibility criteria, application process, and contact information. To ensure real-time updates, financial navigators will maintain weekly contact with these funding agencies throughout the study period.

To support high-fidelity implementation, financial navigators will receive biweekly one-on-one virtual coaching sessions from trainers at TailorMed and NaVectis, focusing on case-based learning, troubleshooting, and documentation practices. In addition, navigators will keep a weekly activity log documenting time spent on core functions, including:

- Patient counseling and communication
- Liaison with charitable and governmental agencies
- Application and follow-up for financial aid programs
- Insurance registration and follow-up
- Number of patients successfully enrolled in financial assistance or insurance programs

This structured workflow will both standardize the navigator role across study sites and provide detailed data for evaluating program fidelity, resource utilization, and implementation cost.

##### Research Staff

**Figure 4:**
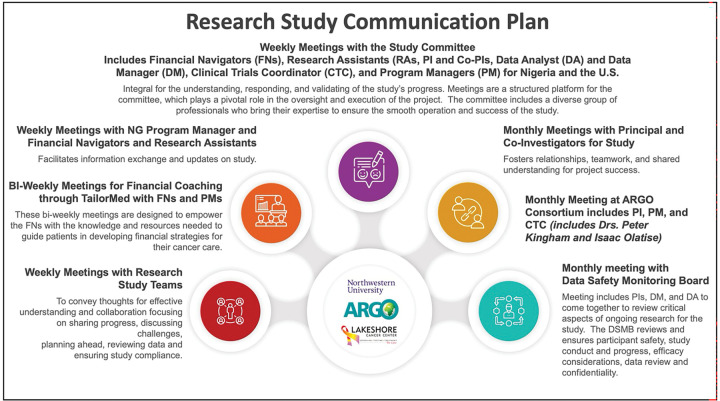
Detailed Communication Plan for COST-FIN

All research staff will undergo mandatory standardized training coordinated through ARGO, focusing on core competencies required for high-quality trial implementation. The training will include modules on the use of REDCap for data capture, secure data storage and management protocols, adherence to Good Clinical Practice (GCP) standards, compliance with standard operating procedures (SOPs), and proper documentation and reporting of adverse events. This centralized training will ensure consistency across sites and promotes data integrity, participant safety, and regulatory compliance. (Figure 4).

### Outcomes {12}

All study outcomes are detailed in Table 1

#### Primary Outcome:

- Financial catastrophe

#### Secondary Outcome:

- Financial distress

#### Exploratory Outcomes:

- Cost of FNP, amount of funding secured through FNP, and the proportion of participants insured.
- Proportion of patients completing treatment, as well as cost-related delays and disruption of care

### Power Calculations – Sample Size Determination {14}

The sample size was calculated using a one-sided 5% alpha level, 80% power, and a 1:1 control versus treatment allocation ratio.

#### Rationale for Sample Size Determination

##### Financial Catastrophe:

The sample size was estimated based on HHI, cost of treatment, and cost savings from FNP. Calculations were repeated for multiple income distributions (for the entire country, Lagos state, and studies from the SW region). The expected average cost of cancer care was derived from two large studies (n >200 patients each) in the SW region and estimated at $4,754 (standard deviation SD = $5,046).^21,55^ The estimate for cost-savings, $2,500, is based on the fixed effect of insurance (HMOS) on out-of-pocket costs and the extent of cost-sharing. Our estimates are based on verified plans that cover cancer patients and do not exclude based on pre-existing conditions. These estimates suggest that approximately 80% of controls and 64% in the intervention arm would experience FC. To detect this difference at a one-sided alpha of 5% using a test for proportions requires 95 participants per arm (190 total).

##### Financial Distress:

Previous data among prostate cancer patients in Nigeria shows a mean COST-FACIT score of 26.5 (SD = 10.08).^56^ Studies on FNPs in the U.S. have reported 7-point FD improvements on average, though these samples had worse baseline scores (COST < 19), so we expect a smaller effect in our sample.^57–59^ We expect an average score of 26.5 (SD = 10.08) in the control arm and 31.5 (SD = 10.08) in the intervention arm. To detect this difference at a one-sided 5% alpha level using a two-sample t-test, and account for an anticipated 20% attrition to the 3-month follow-up questionnaire, requires 60 participants per arm (120 total).

Thus, considering the calculations mentioned above, the recruitment target for this study is 200 participants. Based on breast, prostate, and colorectal cancer patient volume at the two study sites in recent years, we estimate an enrollment period of up to 12 months to recruit 200 eligible participants.

### Participant Recruitment, Randomization, and Blinding {16a, 16b, 16c, 17a and 17b}

#### Recruitment Plan

Both participating study sites maintain active multidisciplinary tumor boards that convene weekly to review newly diagnosed cancer patients. These meetings will serve as a primary source for identifying eligible participants in real time. Additional recruitment opportunities include outpatient clinic appointments, referrals from treating providers, and self-referral through posted study information and community outreach materials.

Eligible patients will be approached in person by trained research assistants during their clinical visits. Research staff will provide study information sheets outlining the purpose of the trial, study procedures, data privacy protections, and the voluntary nature of participation. Patients expressing interest will be invited to proceed with the consent process. After informed consent is obtained, participants will complete a baseline survey, and study data will be entered directly into the secure REDCap database, with routine data quality checks performed by the data management team.

#### Recruitment Procedures

Research assistants, trained in ethical research conduct and Good Clinical Practice (GCP), will be responsible for obtaining informed consent from all participants. The consent form will be reviewed verbally and interactively, with ample opportunity for questions. Once full understanding is confirmed, participants will provide written consent, and an electronic acknowledgment will be documented in REDCap. Participants will receive printed copies of the informed consent form and study brochure for their records.

During the enrollment visit, participants will be informed about:

- The timing and purpose of the baseline survey
- The process and timing of randomization
- The schedule for the 3-month and 6-month follow-up surveys
- Instructions to retain receipts and cost documentation related to cancer care
- Plans for periodic communication via phone calls, SMS, or email from research staff
- Medical record review procedures for abstracting clinical diagnoses and treatment details

When feasible, participants may be scheduled to meet briefly with study staff before or after routine clinical appointments to minimize additional burden. Figure 1b depicts the full study flow, from screening through final follow-up.

#### Randomization

Following informed consent and baseline data collection, participants will be randomized in a 1:1 ratio to either the intervention (FNP) or the routine care arm of the study, within two weeks of enrollment. Randomization will be implemented using the secure, computer-generated blocked randomization scheme in REDCap’s randomization module, with allocation sequences preloaded and access restricted to authorized personnel only. This will ensure rigorous allocation concealment, prevent selection bias, and maintain the integrity of the trial.

To account for potential heterogeneity in financial need and clinical trajectory, randomization will be stratified by:

- Cancer type (i.e., breast, colorectal, or prostate)
- Stage at diagnosis (early versus advanced)
- Study site (LCC versus OAUTHC)

Treatment assignments will be fixed upon randomization. No crossover or re-randomization will be permitted unless a pre-specified interim analysis, conducted once 50% of participants have reached 6-month follow-up, demonstrates clear superiority of the FNP over routine care in the primary or secondary outcomes of reduction in FD or FC. Interim analysis will follow a conservative alpha-spending approach to preserve study power.

Allocation and concealment procedures will be overseen by a designated data manager independent of participant recruitment and clinical care processes.

#### Implementation of Randomization Outcome

Within two weeks of randomization, the study biostatistician will notify the clinical trials coordinator and designated members of the study team for each participant’s group assignment using a secure, study-specific email address. Upon receipt of the assignment, the financial navigators will initiate contact with participants assigned to the intervention arm within one week, beginning the navigation protocol as outlined. This streamlined notification process will ensure timely intervention delivery while maintaining clear communication and documentation across study personnel.

#### Participants Engagement after Randomization

Following enrollment, participants randomized to the intervention arm will be put in contact with a financial navigator for structured support throughout the study period. The baseline questionnaire will be administered by trained research assistants through a brief 10-minute in-person interview, conducted during a routine clinic visit to minimize participant burden.

Financial navigators will then collaborate with each participant to develop a personalized financial plan, tailored to the individual’s financial circumstances, treatment trajectory, and eligibility for available support resources. Navigators will facilitate access to charitable assistance programs, insurance enrollment, medication aid, and other forms of financial relief. These interactions may occur in person, by phone, or through electronic platforms, depending on participant preference and logistical feasibility.

Key engagement metrics will be systematically tracked, including:

- Time spent navigating financial resources
- Timeliness of navigator response to patient needs
- Frequency and mode of patient contact
- Number of successful linkages to support programs

To acknowledge participants’ time and ensure continued engagement, they will receive modest compensation via mobile airtime transfers, which may be used for voice/data communication or converted to offset transportation and logistical costs associated with study participation.

#### Monitoring Participant Recruitment

Trial recruitment progress will be monitored on a weekly basis by the ARGO program manager, the study clinical project manager, and the principal investigator to ensure timely and equitable enrollment. Recruitment data will be reviewed for total accrual, site-specific progress, and representation across cancer types, stages, and sociodemographic strata.

In the event of under-enrollment, particularly among key subpopulations (e.g., rural residents, uninsured patients, or specific cancer types), the research team will implement targeted outreach strategies. These may include:

- Enhanced engagement during tumor board meetings
- Tailored informational materials in local languages
- Focused recruitment through support groups and community partners
- Adjustments to research assistant workflows to prioritize outreach

#### Blinding

Given the nature of the intervention in this trial, it is not feasible to blind patients, financial navigators, or clinical providers to treatment allocation. However, the study will incorporate several strategies to minimize bias. The principal investigator will remain blinded to group assignments throughout data collection and primary analysis to preserve objectivity. Since some co-investigators are practicing clinicians involved in patient care, they will be unblinded to allocation status (FNP versus routine care) by necessity.

Importantly, all primary and secondary outcome assessors, including those conducting data entry, statistical analysis, and qualitative coding, will be blinded to participant allocation. Blinded review of patient records, COST-FACIT scores, and treatment adherence data will help reduce observer and confirmation bias.

### Data Collection Protocol {18a and 18b}

Trained research assistants will conduct structured, questionnaire-based interviews with study participants at four key time points: baseline, 3 months, 6 months, and 12 months post-randomization. Interviews will be conducted in person or via phone, depending on participant accessibility and preference.

#### Baseline Data Collection

The baseline questionnaire will collect comprehensive information across the following domains:

- Demographic parameters (age, sex, education, marital status, employment)
- Socioeconomic status (SES): Measured using the Household Wealth Index, a validated 10-item asset-based tool assessing dwelling characteristics and household possessions to stratify SES into quintiles.^60^
- Clinical history (cancer type, stage, comorbid conditions, treatment modality)
- Financial literacy: Assessed with the Standard and Poor’s Global Financial Literacy Survey.^61^
- FD: Measured using the FACIT-COST instrument
- Health-related quality of life: Evaluated with the 12-item Short Form Survey (SF-12) and 5-level EQ-5D version (EQ-5D-5L) – a 2-page instrument consisting of the EQ-5D descriptive system and the EQ visual analogue scale (EQ VAS) first introduced in 2009.^62,63^
- Food insecurity: Assessed using the Hunger Vital Sign^™^, a validated 2-item screening tool for household food insecurity.^64^

Additionally, at baseline, participants will be asked whether they have experienced economic hardship in the 12 months prior to diagnosis, including inability to pay for food, housing, school fees, or healthcare expenses.

The study will also collect detailed data on timelines of care, including delays in diagnosis and treatment initiation. These metrics are critical to ensure a detailed understanding of the pathways through which FD contributes to advanced-stage presentation and poor outcomes in low-resource settings.

Specifically, the following care delay metrics will be captured:

- Time from symptom onset to first medical consultation
- Time from first consultation to histologic diagnosis
- Time from diagnosis to treatment initiation
- Patient-reported barriers to timely care (e.g., cost, transportation, systemic delays)

These data will be extracted from medical records and patient interviews. Delays will be classified using WHO cancer care continuum benchmarks.

#### Cancer Diagnosis and Treatment Data

Diagnostic and treatment data information will include

- Method of diagnosis
- Cancer stage
- Immunohistochemistry
- Recommended treatment
- Treatments received

Recommended and received treatments will be extracted from medical charts by the research assistants and additionally confirmed by medical oncologists.

#### Out-of-Pocket Costs Data

Out-of-pocket costs will be assessed using a multi-pronged approach to capture the full extent of financial burden related to cancer treatment. Data will be collected in the following three domains:

##### 1. Direct Internal Costs:

Costs incurred at the participating study sites will be abstracted from hospital billing records and institutional financial ledgers.

##### 2. Direct External Costs:

Participants will be asked to submit receipts for services obtained at external facilities (e.g., diagnostic centers, pharmacies, other hospitals), acknowledging the lack of integrated electronic medical records.

##### 3. Indirect Costs:

Structured interviews will be used to estimate ancillary costs including:

- Transportation expenses for patients and caregivers
- Missed workdays due to treatment
- Lost wages, calculated using the national average daily wage multiplied by reported days of work missed
- Opportunity costs of caregiving

Participants will be encouraged to retain receipts for all treatment-related expenses. These will be reviewed and collected by research assistants during scheduled study contacts. To minimize participant burden, designated caregivers or healthcare proxies may assist with record-keeping.

#### Cost-Benefit Data of Financial Navigation Program

To assess the economic efficiency and sustainability of the FNP, a site-specific cost-benefit analysis will be conducted over the study period. The analysis will quantify both the operational costs of program implementation and the financial resources mobilized for patients.

Program costs will include:

- Financial navigator salaries and fringe benefits
- Recruitment and onboarding expenses
- Training costs
- Ongoing coaching and consultation
- Administrative overhead costs associated with scheduling, documentation, and stakeholder engagement

Program benefits will be captured through financial navigator logs and institutional records and will include:

- Charitable grants and non-governmental organizations (NGOs) funding obtained for patient care.
- Successful insurance enrollments and reimbursements (e.g., government schemes, employer-based coverage)
- Medication assistance through pharmaceutical access programs
- Institutional cost savings (e.g., reductions in uncompensated care, charity write-offs, or patient attrition)

### Language-related Issues in Data Collection, and Collection of Biological Specimen {26a, 26b, and 33}

To ensure inclusivity and accuracy in data collection across diverse patient populations, all financial navigators and research assistants will be fluent in English and at least one of the predominant local languages (e.g., Yoruba, Hausa, or Igbo). While standardized data collection instruments will be developed and maintained in English to preserve consistency across sites, participants will be interviewed in their preferred language to optimize comprehension and comfort.

The use of trained interviewers administering structured questionnaires will help minimize literacy-related bias, especially among participants with limited formal education. Interviewers will be trained to use culturally sensitive, non-leading language and neutral tones to avoid introducing interpretation bias. Pre-testing and cognitive interviewing will be conducted during the pilot phase to ensure clarity and appropriateness of all instruments. To maintain data integrity across languages, a standard glossary of key terms will be developed and regularly updated, and inter-rater reliability checks will be periodically conducted across interviewers. No biological specimens will be collected in this study.

### Data Quality Review {19}

Ensuring high-quality data is a central component of this study’s implementation strategy. A designated data quality manager will oversee all aspects of data entry, monitoring, and reporting throughout the duration of the trial.

Key activities will include:

#### Weekly Audits of all REDCap Entries

These audits will ensure:

- ***Completeness***: All required fields are populated or appropriately marked as “Don’t Know,” “Refused,” or “Not Applicable.”
- ***Response accuracy and logic***: Entries will be checked for consistency across fields (e.g., reported income versus SES indicators).
- ***Formatting compliance***: Ensuring that all responses conform to pre-specified formats (e.g., numeric fields, categorical responses).

#### Cost Receipt Reconciliation:

Each submitted treatment-related expense will be reviewed to ensure the total amount and classification (e.g., diagnostic cost, medication, transport) matches the corresponding cost instance form.

#### Data Query Reports:

These reports will be generated weekly and shared with site coordinators, identifying discrepancies or missing values using participant identification (ID) numbers. Timely follow-up and corrections will be tracked and logged.

#### Bi-weekly virtual meetings:

Held via Zoom between the data quality manager and local site teams, they will:

- Address unresolved queries
- Provide feedback on common data entry issues
- Harmonize documentation and troubleshoot REDCap platform use

Additionally, inter-rater reliability checks will be performed quarterly to ensure consistency across research assistants, particularly for abstracted chart data and cost categorization.

This continuous quality assurance process will uphold the rigor and credibility of the trial’s outcome analyses and support future scalability of the FNP intervention in diverse settings.

### Timeline for Assessment of Study Outcomes {13}

Detailed data on demographic, social and clinical characteristics, prescribed treatments, incurred costs and financial aid secured, for assessment of outcomes, will be collected with the help of structured interviews at baseline, 3 months, 6 months, and 12 months post-randomization (Figure 2).

### Post Trial Care {30}

Following trial completion, all participants will continue to receive cancer care as determined by shared clinical decision-making with their oncology care teams. Treatment decisions will be guided by the patient’s clinical condition, preferences, and standard protocols at each institution.

In alignment with ethical obligations for research in low-resource settings, the following support will be provided:

#### Intervention arm:

Participants will receive a final debriefing session with their financial navigator, including a personalized financial plan and documentation of all resources accessed during the study.

#### Control arm:

Participants will be given a comprehensive resource packet outlining available financial assistance programs, insurance enrollment support, and referral pathways to social workers or institutional navigation services.

### Statistical Analysis Plan {20a, 20b and 20c}

Data summaries by trial arm will include measures of central tendency (mean, standard deviation, median, quartiles), as appropriate. Two-sample t-test, Wilcoxon’s, or tests for proportions will be employed to compare groups, as appropriate.

#### Primary Outcome – Financial Catastrophe:

To assess FC, the sum of all direct and indirect out-of-pocket costs of cancer-related treatments will be represented as a proportion of their HHI, HHE, and HSE. The risk of FC for patients who stop treatment due to the inability to pay will also be estimated.

Financial catastrophe (FC) will be analyzed as a binary outcome, defined as health expenditures exceeding 10% of household income, 25% of household expenditure, or 40% of non-subsistence expenditure, or discontinuation of treatment due to inability to pay. The primary comparison will be between the intervention and control arms to evaluate the effect of the financial navigation program.

We will first conduct a summary comparison of the incidence of financial catastrophe between the arms using a one-sided test of proportions at an alpha level of 0.05. This will provide a straightforward estimate of the unadjusted effect of the intervention.

To adjust for baseline differences and account for clustering or repeated measures, we will additionally employ a generalized linear mixed-effects model (GLMM) with a logit link. This model will include fixed effects for treatment arm, cancer type, cancer stage, age, sex, and socioeconomic status, as well as a random intercept for site to account for potential between-site heterogeneity. If FC is measured at multiple time points (e.g., baseline, 6 months, 12 months), we will also include a random intercept for participant to account for within-subject correlation over time. This modeling approach provides a robust estimate of the intervention effect while accounting for potential clustering and intra-individual correlation in the data.

Results will be presented as odds ratios with 95% confidence intervals. Sensitivity analyses will be conducted to evaluate the robustness of findings, including complete-case versus imputed datasets, and models with alternative thresholds for defining financial catastrophe.

#### Secondary Outcome – Financial Distress:

As detailed above, FD will be measured with the FACIT-COST instrument, which is based on 12 Likert-scale questions and has been validated for use in cancer patients and applied in Nigeria.^50,56,65^

To evaluate the effect of the financial navigation program on financial distress over time, a linear mixed-effects model (LMM) will be used. This model will estimate the change in FACIT-COST scores from baseline to follow-up (e.g., baseline, 3 months, 6 months, 12 months), with fixed effects for study arm, time, and an arm-by-time interaction. Random intercepts will be specified at the individual level to account for within-subject correlation.

The LMM will allow us to estimate both the average trajectory of financial distress in each arm and the differential rate of improvement attributable to the intervention. The key parameter of interest is the arm-by-time interaction coefficient, which represents the average additional change in financial distress in the FNP group relative to the control group.

Summary statistics will also be reported, including mean (SD), median (IQR), and range of FACIT-COST scores by study arm at different follow-up timepoints. For participants who have not yet reached the 6-month time point, their most recent available score (e.g., at 3 months) may be used in a sensitivity analysis to maximize available data.

#### Exploratory Secondary Analyses

##### Cost-related non-adherence:

To evaluate the impact of FNP on cost-related non-adherence, data on delays in presentation, treatment initiation, and deviation from recommended treatment will be collected via structured participant and treating medical oncologist interviews at six months. For each treatment modality, the proportion of participants not pursuing the prescribed treatment will be determined, including whether the decision not to pursue the recommendation was cost-related. Multivariable logistic regression modelling with relevant variables will be used to assess non-adherence to prescribed treatment between the intervention and control groups.

##### Cost-Benefit Analysis:

At each study site, a Benefit-Cost Ratio (BCR) will be derived by dividing the total FNP-driven financial savings/benefits accrued by the total program costs. Benefits will include direct monetary savings secured by navigators on behalf of patients (e.g., insurance reimbursements, charitable funds, out-of-pocket cost reductions) over the study period. Costs will reflect navigator personnel time (converted to salary equivalents), administrative overhead, training, and other operational costs necessary to deliver the FNP. A BCR ratio greater than 1^66,67^ will indicate overall cost-effectiveness of the FNP program in the unique settings at both study sites and provide strong justification for the adoption of similar programs in resource-limited settings.

These analyses will help characterize the range of BCRs under plausible real-world variations and inform sustainability planning in other low-resource settings.

#### Interim Analysis and Crossover from Control to Intervention Arm {21b}

An **interim analysis** will be conducted when **50% of the target sample** has completed at least **six months of follow-up**, projected to occur approximately **12 months after study initiation**. This analysis is designed to evaluate early evidence of efficacy for the FNP and guide ethical decisions regarding continued randomization.

Based on the **pre-specified power calculations** for the interim sample, the following will be considered statistically significant at a **one-sided α = 0.05** threshold.:

- A **≥23% absolute reduction** in the incidence of **FC.**
- A **≥5.6-point improvement** in **FD** as measured by the COST-FACIT score

The **Data and Safety Monitoring Board (DSMB)** will review unblinded interim results to determine whether there is **compelling evidence of benefit**. If such evidence is observed:

- All enrolled participants in the **control arm** will be offered **crossover access to the FNP**.
- Subsequent participants may be offered enrollment into the intervention arm only, pending IRB approval and ethical review.

Despite crossover, **all participants will be followed** for **12 months post-randomization** for endpoint ascertainment. This ensures the completeness of outcome data for primary, secondary, and exploratory outcomes, including FC, FD, treatment adherence, quality of life, and cost metrics.

The interim analysis will be conducted by an **independent biostatistician** unaffiliated with recruitment or care delivery, using **intention-to-treat principles**.

#### Loss to Follow-up and Missing Data

We estimate 20% cancer-related mortality within the 12-month follow-up period among participants, based on cancer survival data in Nigeria.^4,7,9^ Loss-to-follow-up for reasons other than death (stopped treatment, transfer of care, or withdrawal) is estimated at around 10% over 12 months,^7,9,37^ resulting in a 70% completion rate. Given the minimal risk of the intervention and the perceived benefits, a minimal intervention-related drop-out is expected.

Data on reasons for dropout will be collected. Informative dropout may occur if participants at higher risk of FC or FD discontinue participation. If it appears plausible, additional analyses addressing this aspect will be conducted.^68–70^

Cross-tabulations of proportions of missing data on all baseline characteristics and primary outcome measures will be provided. To investigate systematic differences between participants lost-to-follow-up and those still in-study, the missing data mechanism will be modeled as a function of baseline covariates, including study arm. Various multiple imputation strategies will be considered, as needed.

### Trial Oversight, Safety Monitoring, Protocol Modifications, and Dissemination of Findings and Data

#### Confidentiality {27}

All personally identifiable participant data will be held in strict confidence within the research team, and no identifiable information will be released to any unauthorized third parties without prior written approval of the sponsoring agencies.

#### Data Safety and Monitoring Plan {21a, 22 and 23}

This study is considered minimal risk and poses little to no harm to participants. Given the sensitive nature of the sociodemographic questions, the interviews will be conducted in a private setting to ensure comfort and privacy for participants.

The only anticipated risk to study participants is the loss of data confidentiality. To minimize this, all data will be collected and stored in a secure, password-protected REDCap database hosted at ARGO on a 21 CFR Part 11 server maintained by Northwestern University Information Technology and ARGO. Access to the database will be centrally managed by appropriately trained personnel, restricted to those with a legitimate need, and periodically reviewed by the data analyst. Use of personal tablets or laptops will be strictly prohibited except through secure VPN connections. No participant data may not be downloaded to personal machines. Research records will be retained for at least six years after the completion of the research.

COST-FIN will be overseen by an independent DSMB, convening regularly based on study accrual. The DSMB will include implementation scientists, epidemiologists, health economists, biostatistics, community participatory researchers, and ethicists, with representation from the United States and Sub-Saharan Africa. It reviews study progress, enrollment, attrition, safety, regulatory compliance, and data integrity. Any concerns raised during DSMB mandate a formal response in writing until DSMB members are satisfied. Summaries and minutes of these meetings will be documented and made available for inspection as requested or required by official regulatory bodies responsible for the safety of human subjects and the integrity of trial data.

The principal investigator will promptly review and respond to any reported participant concerns or adverse events. In the event of data breach, the principal investigator will notify the DSMB, Northwestern University IRB, University of Lagos and OAUTHC IRB, and the National Institutes of Health (NIH) within two business days of discovery of the event.

#### Dissemination Plan {31a}

The results of COST-FIN will be disseminated through peer-reviewed publications, direct communication with key stakeholders at all trial sites, and by presentation at national and international scientific meetings. This multi-platform dissemination strategy will ensure that the study’s impact is extended across academic, clinical, and policy-making communities in Nigeria and beyond.

#### Protocol Access, Data Sharing and Archiving {31c}

The COST-FIN study will comply with the NIH Public Access Policy. The scientific data and metadata will be archived in the Clinicaltrials.gov repository, operated by the U.S. National Library of Medicine (NLM), and will be identifiable through a unique identification code assigned by the registry. Following study conclusion, the data will be uploaded to Clinicaltrials.gov and made available to the wider research community indefinitely in accordance with the informed consent provisions and institutional certification. All shared datasets will be de-identified according to the Safe Harbor Method to protect the privacy, rights, and confidentiality of study participants. Full trial protocol and statistical code may also be requested by researchers and other relevant bodies by contacting the study principal investigator.

#### Communication of Protocol Amendments to Relevant Parties {25}

The study principal investigator will seek approval from all relevant IRBs in case of any amendments. The rationale for the proposed amendments will be detailed.

## DISCUSSION

This clinical trial, the first of its kind in Sub-Saharan Africa, aims to investigate the impact of a structured FNP on cancer-care related financial toxicity, potentially reducing barriers to cancer care and improving treatment adherence in Nigeria. Given the paucity of evidence on FNPs in low-resource settings, particularly in Sub-Saharan Africa, this study will generate landmark data on the feasibility and effectiveness of FNPs in mitigating financial risk, identifying cost-related factors influencing treatment outcomes, and assessing the financial cost-benefit profile of FNPs.

Our previous studies in breast cancer and colorectal cancer patients at LCC in Nigeria demonstrated that an overwhelming majority of patients faced FC. This was especially true in patients undergoing multi-modality treatment, e.g., 100% of breast cancer patients in this subgroup risked FC, which is often the standard of care in modern cancer treatment protocols.^24^ Yezefski *et al* demonstrated in a study of around 11,000 cancer patients that FNPs not only significantly decrease the out-of-pocket expenses for cancer care but also have major financial incentives for treating facilities.^41^ Furthermore, access to information regarding estimated costs and potential funding avenues can also reduce the patient and family members’ mental health and mitigate anxiety, improving overall well-being. Finally, FNPs not only reduce financial toxicity and improve mental health related to cancer care in resource-constrained settings but also improve treatment adherence and thus long-term cancer outcomes. In light of these findings, the results of COST-FIN could have major system-wide implications for cancer care in Nigeria and the wider region. From an implementation standpoint, previous data have demonstrated that FNPs can be successfully incorporated into cancer care at any point along the cancer care continuum, making FNPs especially effective as a strategy to minimize financial toxicity.

Besides its landmark nature, the major strength of this study and intervention lies in its easy-to-replicate model that may be adapted for use and be scaled in a variety of other settings in Sub-Saharan Africa or other LMICs. Furthermore, the multi-center design, including both a private (LCC) and a public facility (OAUTHC) in two separate states (Lagos and Osun states) and inclusion of several common cancers (breast, prostate or colorectal cancer) increases patient and disease heterogeneity, and thus the generalizability of our findings.

In the near future, the wider economic implications of cancer care-related FC and FD, premature mortality from suboptimal treatment, decreasing economic productivity, and increasing unemployment will become increasingly important given the rising burden of cancer in LMICs. This trial will provide timely and crucial evidence to support the implementation of FNPs in cancer care in LMICs, specifically in Sub-Saharan Africa which has the highest burden of premature deaths. Therefore, its findings and their potential implementation could have national implications for beyond the health system.

## Supplementary Material

Supplementary Files

This is a list of supplementary files associated with this preprint. Click to download.

• COSTFINTrialCONSORT2025editablechecklist.docx

• SPIRITFIGURE71.docx

SPIRIT FIGURE

Spirit figure is available in the Supplementary Files section.

## Figures and Tables

**Table 1 :** Aims and Outcomes of COST-FIN Study

Aims	Outcomes	
To evaluate the effect of the financial navigation program on financial catastrophe in patients with cancer.	**Primary**	• Financial catastrophe Defined by the World Health Organization as out-of-pocket expenses that exceed 10% of household income (HHI), 25% of household expenditures (HHE) or 40% of non-subsistence expenditures (HSE).
To determine whether the financial navigation program reduces financial distress for patients with breast, prostate and colorectal cancer.	**Secondary**	• Financial Distress Measured by COST-FACIT (Comprehensive Score for financial Toxicity-Functional Assessment of Chronic Illness Therapy), a validated tool for measuring financial distress in individuals with cancer.^36^ It comprises 11 statements with a 5-point response, and lower scores representing worse financial toxicity
To determine if financial navigation program is associated with cost-related nonadherence to cancer care for patients with breast, prostate and colorectal cancer.	**Exploratory**	• Cost of financial navigation program • Total amount of funding secured through financial navigation program • The proportion of participants insured. • Proportion of patients completing treatment • Cost-related non-adherence • Delay in presentation and treatment initiation • Deviation from recommended treatment

## Data Availability

This study will comply with the NIH Data Sharing Policy and Policy on the Dissemination of NIH-Funded Clinical Trial Information and the Clinical Trials Registration and Results Information Submission rule. Thus, this trial has been registered at ClinicalTrials.gov, and the results of this trial will be submitted to ClinicalTrials.gov. Study results will be published in peer-reviewed journals. Study data may be requested by contacting the principal investigator.
